# COVID-SGIS: A Smart Tool for Dynamic Monitoring and Temporal Forecasting of Covid-19

**DOI:** 10.3389/fpubh.2020.580815

**Published:** 2020-11-17

**Authors:** Clarisse Lins de Lima, Cecilia Cordeiro da Silva, Ana Clara Gomes da Silva, Eduardo Luiz Silva, Gabriel Souza Marques, Lucas Job Brito de Araújo, Luiz Antônio Albuquerque Júnior, Samuel Barbosa Jatobá de Souza, Maíra Araújo de Santana, Juliana Carneiro Gomes, Valter Augusto de Freitas Barbosa, Anwar Musah, Patty Kostkova, Wellington Pinheiro dos Santos, Abel Guilhermino da Silva Filho

**Affiliations:** ^1^Polytechnique School of the University of Pernambuco, Poli-UPE, Recife, Brazil; ^2^Center for Informatics, Federal University of Pernambuco, Recife, Brazil; ^3^Department of Biomedical Engineering, Federal University of Pernambuco, Recife, Brazil; ^4^Institute for Risk and Disaster Reduction, University College London, London, United Kingdom

**Keywords:** SARS-CoV-2 spread forecast, intelligent forecasting systems, infectious diseases, dynamic forecasting systems, Covid-19 forecasting

## Abstract

**Background:** The global burden of the new coronavirus SARS-CoV-2 is increasing at an unprecedented rate. The current spread of Covid-19 in Brazil is problematic causing a huge public health burden to its population and national health-care service. To evaluate strategies for alleviating such problems, it is necessary to forecast the number of cases and deaths in order to aid the stakeholders in the process of making decisions against the disease. We propose a novel system for real-time forecast of the cumulative cases of Covid-19 in Brazil.

**Methods:** We developed the novel COVID-SGIS application for the real-time surveillance, forecast and spatial visualization of Covid-19 for Brazil. This system captures routinely reported Covid-19 information from 27 federative units from the Brazil.io database. It utilizes all Covid-19 confirmed case data that have been notified through the National Notification System, from March to May 2020. Time series ARIMA models were integrated for the forecast of cumulative number of Covid-19 cases and deaths. These include 6-days forecasts as graphical outputs for each federative unit in Brazil, separately, with its corresponding 95% CI for statistical significance. In addition, a worst and best scenarios are presented.

**Results:** The following federative units (out of 27) were flagged by our ARIMA models showing statistically significant increasing temporal patterns of Covid-19 cases during the specified day-to-day period: Bahia, Maranhão, Piauí, Rio Grande do Norte, Amapá, Rondônia, where their day-to-day forecasts were within the 95% CI limits. Equally, the same findings were observed for Espírito Santo, Minas Gerais, Paraná, and Santa Catarina. The overall percentage error between the forecasted values and the actual values varied between 2.56 and 6.50%. For the days when the forecasts fell outside the forecast interval, the percentage errors in relation to the worst case scenario were below 5%.

**Conclusion:** The proposed method for dynamic forecasting may be used to guide social policies and plan direct interventions in a cost-effective, concise, and robust manner. This novel tools can play an important role for guiding the course of action against the Covid-19 pandemic for Brazil and country neighbors in South America.

## 1. Introduction

The world faces a new pandemic that is spreading at an alarming rate. The ongoing outbreak is caused by an acute infectious disease known as severe acute respiratory syndrome coronavirus (SARS-CoV-2) which is responsible for the current coronavirus disease 2019 (Covid-19) pandemic. The probable origins of SARS-CoV-2 are from the Pangolins, a mammalian animal of the order Pholidota (1). The SARS-CoV-2 has a high transmission rate, within a short time period between December 2019 and May 2020, more than 4.7 million people were infected in 216 countries (2). The acute symptoms for Covid-19 includes fever, cough, sore throat, fatigue, and shortness of breath (3); however, in some cases if the symptoms are not managed, it can develop into severe pneumonia which, in turn, leads to critical conditions, such as sepsis or acute respiratory distress syndrome which can be fatal. As of May 2020, the Covid-19 has claim more than 317,000 lives and this figure shall continue to rise in the coming months (2).

The gold standard test for diagnosing Covid-19 is the Reverse Transcription Polymerase Chain Reaction (RT-PCR) with DNA sequencing and identification (4). Nevertheless, the RT-PCR needs several hours to return a result (4). While the RT-PCR identifies directly the presence or absence of the virus, on the other hand, the rapid test may sometimes be non-specific. They detect the serological evidence of recent infection based on the presence of antibodies. However, the production of antibodies starts after some days, or even weeks after the infection (5). Besides that, this kind of tests could recognize antigens of other viruses; for example, influenza and other coronaviruses (6). What compromise the test's accuracy; for example, are tests based on IgM/IgG antibodies which were realized in samples collected in the first week of the illness have 18.8% sensitivity and 77.8% specificity (7).

Currently, there is no vaccine nor a specific treatment for curing Covid-19. In a home or hospital settings, one can only ease the symptoms through the course of the infection until s/he recovers. The best strategy to decrease its transmission within a population is to quarantine those infected with Covid-19, and to encourage the practice of social and physical distancing on a mass scale. This course of action provides a much safer and practical approach for limiting contact between the infected and those at risk of infection (8). This course of action, and in addition to, the cancellation or postponement of large public events as well as deferment of non-essential activities on a mass scale has significantly helped in controlling the spread of the virus as observed in China and elsewhere. Social isolation and quarantine also prevents people with asymptomatic infection from spreading the virus (8, 9). Other works also show positive results in the reduction of its transmission with social isolation in effect in countries, such as Italy (9), Switzerland (10), and Brazil (11).

One of the approaches used to combat diseases include the forecast of the number of cases based on the behavior of past events. In the case of arboviruses, for example, the time series of the number of cases and climatic variables are used to forecast future behaviors (12–14). Considering the SARS-CoV-2, forecasting the pandemic trends could be crucial to avoid the virus spreading. Many mathematical models, such as Susceptible-Infected-Removed (SIR) and its variations have being used to forecast Covid-19 pandemic trends (15–20).

Therefore, the main goal of this research is to present a methodology for monitoring and to provide forecasts of cases and deaths of Covid-19 for each federative units of Brazil. This research relies on an interdisciplinary approach that brings together digital health, statistical modeling, GIS and computer sciences to create a system which comprise of a dynamic web application interfaced with multiple databases reporting incident cases (and deaths) of Covid-19 in Brazil. This tool will churn the information to provide forecasts on a national and state-level. The Autoregressive Integrated Moving Average (ARIMA) method (21) was utilized for this purpose. The reasons for choosing the ARIMA model: (1) it provides the user with dynamic forecasts for a daily basis through model training (per day with a maximum window of 3 days); and (2) its flexibility and robustness in terms of providing user three important outputs [i.e., the forecasted number future cases with their corresponding 95% confidence interval (CI) as well as both best and worst case scenarios].

This paper is organized as follows: in section 2, we provide a brief discussion about previous studies that used mathematical models to forecast trends of Covid-19 pandemic. In section 3, we describe our proposed method and review its theoretical concepts. In section 4, we present the analysis of the data and the results. Discussions are delivered in section 5, and finally, conclusions are drawn in section 6.

## 2. Related Works

**Figure 1:**
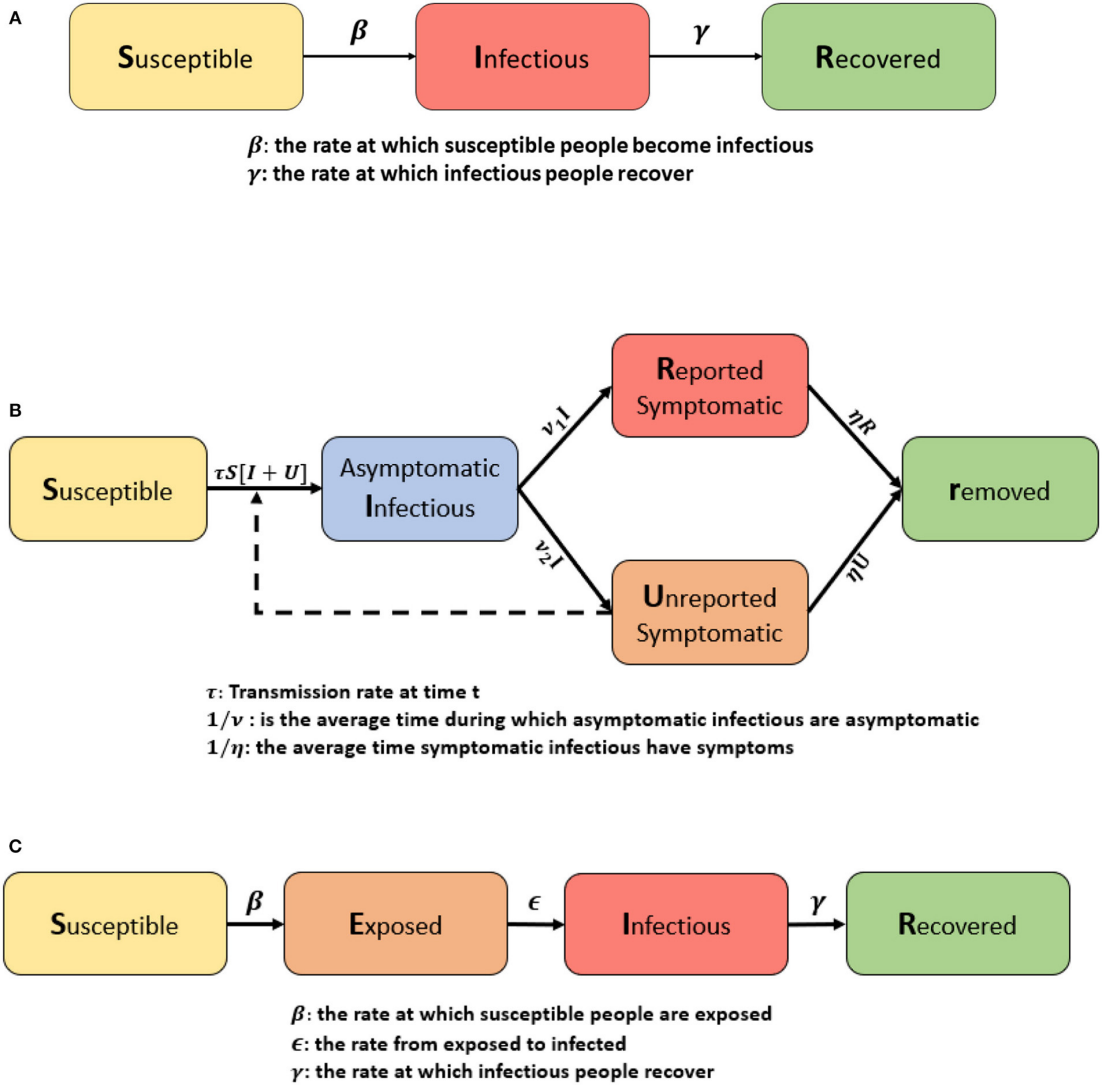
**(A)** Flow chart of the SIR model. The classic SIR model considers three classes: Susceptible, Infectious, and Recovered. β means the rate at which susceptible people can become infectious, and γ is the rate at which infectious people recover. **(B)** Model flow chart adapted from Liu et al. (17). They proposed a modified SIR model by adding asymptomatic infectous people and by dividing symptomatic cases into two classes: reported and unreported. **(C)** Flow chart of the SEIR model. It is composed by four individual classes: Susceptible, Exposed to the virus, Infected and Removed. In contrast to the SIR model, β is the rate at which susceptible people become exposed, ϵ is the rate from exposed to infected, and γ is the rate at which infectious people recover.

Several forecasting models for Covid-19 have emerged around the world. Due to many uncertainties surrounding the behavior of the virus, these models have guided the development of public health strategies and the application of policies that promote social isolation. Given this scenario, several studies have sought to adapt conventional epidemiological models to forecast this pandemic trend (15); for instance, an extended Susceptible-Infected-Removed (eSIR) model was used to forecast epidemics trends in Italy and compared with Hunan (China) due to its similarity in population size and structure. The eSIR model is a version of the classical Susceptible-Infected-Removed (SIR) model. The SIR model uses three different compartments: Susceptible (S), Infected (I), and Recovered (R). This model was presented by Kermack and McKendrick (22), by Britton (23) in his book entitled “Essential Mathematical Biology,” and by Brauer et al. (24) in the book “Mathematical Epidemiology.” The SIR model is used to investigate diseases, such as measles and chickenpox. This model is represented by the following equations:

(1) $$\begin{array}{l} \frac{d}{dt}S=-\beta SI, \end{array}$$

(2) $$\begin{array}{l} \frac{d}{dt}I=\beta SI-\gamma I, \end{array}$$

(3) $$\begin{array}{l} \frac{d}{dt}R=\gamma I, \end{array}$$

where S(t) is the population of susceptible individuals, I(t) is the symptomatic infected individuals, R(t) is the recovered individuals with immunity, β is the contact rate between susceptible and infected individuals, and γ is the transfer rate from I to R. The diagram in Figure 1A illustrates the compartments of the SIR model.

It should be noteworthy that while a SIR model uses a constant transmission rate. The eSIR can account for time-varying transmission rates in the population, and in addition, use time-varying parameters for isolation measures. Wangping et al. (15) used the eSIR forecast the reproduction number (R0) for Covid-19 in Hunan and Italy which was estimated as 3.15 (95% CI: 1.71–5.21) and 4.10 (95% CI: 2.15–6.77), respectively. Due to the discrepancy between these two R0 values, the authors concluded that the results needs to be confirmed in further studies.

In a similar manner, other models, like Bastos and Cajueiro (16), that are akin to SIR have been developed purposefully for forecasting the evolution of Covid-19 in Brazil from February 25, 2020 to March 30, 2020. Data were provided by the Brazilian Ministry of Health. For this purpose, the authors used a modified SIR model in order to create two versions: (1) SID (Susceptible-Infected-Dead) and (2) SIASD (Susceptible-Infected-Asymptomatic-Symptomatic-Dead). In the first version, the authors modified the original model in order to estimate the proportion of individuals who dies from Covid-19. This new parameter also changes the total number of individuals in the population. On the other hand, the second version seeks to improve the SID model. It considers that a relevant part of the population is infected, but asymptomatic. In the SIASD model, the variable of infecteds is divided into two groups accordingly—symptomatic and asymptomatic. In addition, the authors modified the transmission factor which takes into account the effects of the social distancing policies adopted during the selected period. Additionally, this parameter allows the model to evaluate the effectiveness of the measures adopted. In order to estimate the parameters of the models, the work sought to minimize the quadratic error between the real data and the estimated values. The curves were constructed with a 95% confidence interval (95% CI). The authors also considered the underreporting of cases, due to the lack of tests and the government's recommendation to test only patients with severe symptoms. Finally, both models indicated that social distancing policies were able to minimize contagion. It was also possible to conclude that the policies adopted for a short period bought sometime to postpone the peak of the pandemic. Thus, the authors indicated the dates considered to be the optimal for ending the quarantine period. In particular, the SIASD model forecasted a greater number of infections than the SID model. It also indicated a lower peak for symptomatic patients who, in turn, are need of urgent medical attention.

A recent study from China proposed a modified SIR model to forecast the cumulative number of cases of Covid-19 (17). In this example, the model defined by series of five differential equations depicted in a flowchart shown in Figure 1B.

(4) $$\begin{array}{l} S^{\prime }\left(t\right)=-\tau \left(t\right)S\left(t\right)\left[I\left(t\right)+U\left(t\right)\right] \end{array}$$

(5) $$\begin{array}{l} I^{\prime }\left(t\right)=\tau \left(t\right)S\left(t\right)\left[I\left(t\right)+U\left(t\right)\right]-\nu I\left(t\right) \end{array}$$

(6) $$\begin{array}{l} R^{\prime }\left(t\right)=\nu_{1}I\left(t\right)-\eta R\left(t\right) \end{array}$$

(7) $$\begin{array}{l} U^{\prime }\left(t\right)=\nu_{2}I\left(t\right)-\eta U\left(t\right) \end{array}$$

(8) $$\begin{array}{l} r^{\prime }\left(t\right)=\eta R\left(t\right)+\eta U\left(t\right) \end{array}$$

This model formation represents the following: *S*(*t*) is the number of individuals susceptible to infection at time t, *I*(*t*) is the number of asymptomatic infectious individuals at time t, *R*(*t*) is the number of reported symptomatic infectious individuals at time t, *U*(*t*) is the number of unreported symptomatic infectious individuals at time t, τ(*t*) is the transmission rate at time t, $\frac{1}{\nu }$ is the average time during which asymptomatic infectious are asymptomatic, and $\frac{1}{\eta }$ is the average time symptomatic infectious have symptoms.

This model provides information on the number of both asymptomatic and symptomatic infected individuals. It estimates the amount of reported and unreported cases from the symptomatic group. From this study, the authors found that the estimation of unreported cases is extremely important to understand the severity of Covid-19. They also showed the importance of considering the asymptomatic infectious cases for the estimation of disease transmission rate. In these findings, the authors demonstrated the merits of implementing strict control measures by the government, and tighten them so as to decrease the transmission burden.

Similar research utilize a Susceptible-Infectious-Recovered-Dead (SIRD) model to forecast the basic reproduction number (*R*_0_) of SARS-Cov-2 and the daily rates of infection mortality and recovery (18). The *R*_0_ measures the average number of secondary cases that has resulted from an index infectious case. From the *R*_0_ value, a system may forecast the spreadability of an infectious disease. This approach was based on the daily available data of new confirmed cases in Hubei province, China, and was described by the following equations:

(9) $$\begin{array}{l} S\left(t\right)=S\left(t-1\right)-\frac{\alpha }{N}S\left(t-1\right)I\left(t-1\right),\\ I\left(t\right)=I\left(t-1\right)+\frac{\alpha }{N}S\left(t-1\right)I\left(t-1\right) \end{array}$$

(10) $$\begin{array}{l} -\beta I\left(t-1\right)-\gamma I\left(t-1\right), \end{array}$$

(11) $$\begin{array}{l} R\left(t\right)=R\left(t-1\right)+\beta I\left(t-1\right), \end{array}$$

(12) $$\begin{array}{l} D\left(t\right)=D\left(t-1\right)+\gamma I\left(t-1\right), \end{array}$$

The model formation for the above equations are represented as follows: *S*(*t*) is the number of susceptible individuals at time t, *I*(*t*) is the number of infected individuals at time t, *R*(*t*) is the number of recovered individuals at time t, *D*(*t*) is the number of dead individuals at time t, α is the estimation of the infection rate, β is the estimation of the recovery rate, γ is the estimation of the mortality rate, and *N* is the population size.

This model fits the behavior of Covid-19 in Hubei which enables the estimation of key epidemiological parameters. The approach was successful in forecasting the spread of Covid-19 in China. Nevertheless, the SIRD model has some limitations in terms of its inability to include important factors that impacts disease dynamics, such as the effect of the incubation period, demographical characteristics of the population and the effects of restrictive policies. The authors acknowledge point the urgent need for using data in their derived from validated tests. The inclusion of these factors are crucial for building a more accurate and robust forecast models—since they are the key for limiting any residual confounding that may arise from the results, as well as building a more realistic picture for a deterministic point of view.

On the other hand, as proposed by Yang et al. (19) who use a modified Susceptible-Exposed-Infectious-Removed (SEIR) model jointly with the Artificial Intelligence method known as the Long-Short-Term-Memory (LSTM), to forecast epidemics trend of Covid-19 in China under public health interventions. SEIR model is another conventional forecast model, which is composed of four individual classes: Susceptible, Exposed to the virus or in the latent period, Infected and Removed. This model was first introduced by Kermack and McKendrick (22) and is used to understand illnesses like influenza. SEIR model uses the following equation system:

(13) $$\begin{array}{l} \frac{dS}{dt}=-\beta IS, \end{array}$$

(14) $$\begin{array}{l} \frac{dE}{dt}=\beta IS-\epsilon E, \end{array}$$

(15) $$\begin{array}{l} \frac{dI}{dt}=\epsilon E-\gamma I, \end{array}$$

(16) $$\begin{array}{l} \frac{dR}{dt}=\gamma I-\lambda R, \end{array}$$

where S(t) is the population susceptible individuals, E(t) represents the individuals exposed to the disease or in a latent period, I(t) is the symptomatic infected individuals, R(t) is the recovered individuals with immunity, β is the contact rate between susceptible and exposed, ϵ is the transfer rate from class E to I, and γ is the transfer rate from I to R. The diagram in Figure 1C explains the SEIR model.

Yang et al. (19) modified the SEIR model by including the parameters: move-in, move-out, and the contact rate before and after the implementation of control policies. They used the recurrent neural network Long-Short-Term-Memory (LSTM) to corroborate their model forecasts. LSTM was trained on the 2003 SARS epidemic statistics, with available cases between April and June of 2003. They forecasted that the disease in China will peak in late February and end in late April by a combination of their methods. However, using a deep network like LSTM can trigger challenges. The main one is the high memory consumption, since it stores past states, thus making a multi-user application unfeasible (25). In this sense, LSTM is not suitable for client-server applications, such as the one proposed in this study.

In contrast to previous works (20), the authors made a comparative analysis of machine learning (ML) techniques for forecasting the coronavirus outbreak. The authors argue that more traditional models, such as SIR and SEIR, are insufficient to model the pandemic. The reasons are the quarantine and social distance policies applied by many governments in an iterative way, and the lack of data that reveals the real scenario, since the reported data do not actually correspond to the number of infected people. These factors impose great limits on the generalization ability of these conventional models. Thus, the work explores ML techniques to find the best model that estimates time-series data. Data were collected on the worldometers website in five countries: Italy, Germany, Iran, USA and China, corresponding to a period of 30 days. Initially, simple mathematical models were tested (i.e., Logistic, Logarithmic, Quaddratic, Compound, Power, and exponential). In order to estimate the parameters, three optimization algorithms were tested: Genetic Algorithm (GA), Particle Swarm Optimization (PSO), and the Gray Wolf (GWO) optimizer. Considering the processing time, the Root Mean Standard Error (RMSE) and the correlation coefficient as evaluation metrics, the GWO optimizer presented the best results, while the logistic model showed the smallest errors in the forecast of the Covid-19 outbreak for the five countries. However, these models showed low accuracy and low generalization capacity which lacked the ability to properly extrapolate the data beyond 30 days. In the following, two ML methods were introduced for time-series forecast: MultiLayer Perceptron (MLP) and adaptive network-based fuzzy inference system (ANFIS). Both were tested in two different scenarios: Scenario 1 with the data processed weekly; and Scenario 2, with daily sampling of consecutive days. In the case of MLP, they tested 8, 12, and 16 neurons. For ANFIS, the membership function types were Tri, Trap, and Gauss. The comparison between analytical and intelligent models indicated that MLP presented better results in both scenarios, being able to achieve a good approximation with the real data. Long-term data (up to 150 days) were also extrapolated, which reported an outbreak progression. Finally, the authors concluded that ML techniques can be useful in forecasting the Covid-19 pandemic, being able to overcome challenges found in traditional models.

Although the works presented in this section have shown good results, the models using equations require that we estimate a certain number of parameters. It can be a bias for the construction of the model. Moreover, mathematical models are insufficient to analyze the randomness of epidemics and they are also difficult to generalize (26). In addition, as each state has its own dynamics, it makes difficult to implement these models in the proposed tool. ARIMA models, in turn, are good tools for modeling time series because they can capture their changing trends, periodic changes and random distortions in time series. Besides, ARIMA models have been applied in several areas of health due to its simplicity and fast applicability. According to the survey carried out by Ceylan (26), ARIMA models have been used to forecast epidemics, such as dengue, tuberculosis, and hemorrhagic fever, for example.

## 3. Materials and Methods

### 3.1. Proposed Method

**Figure 2:**
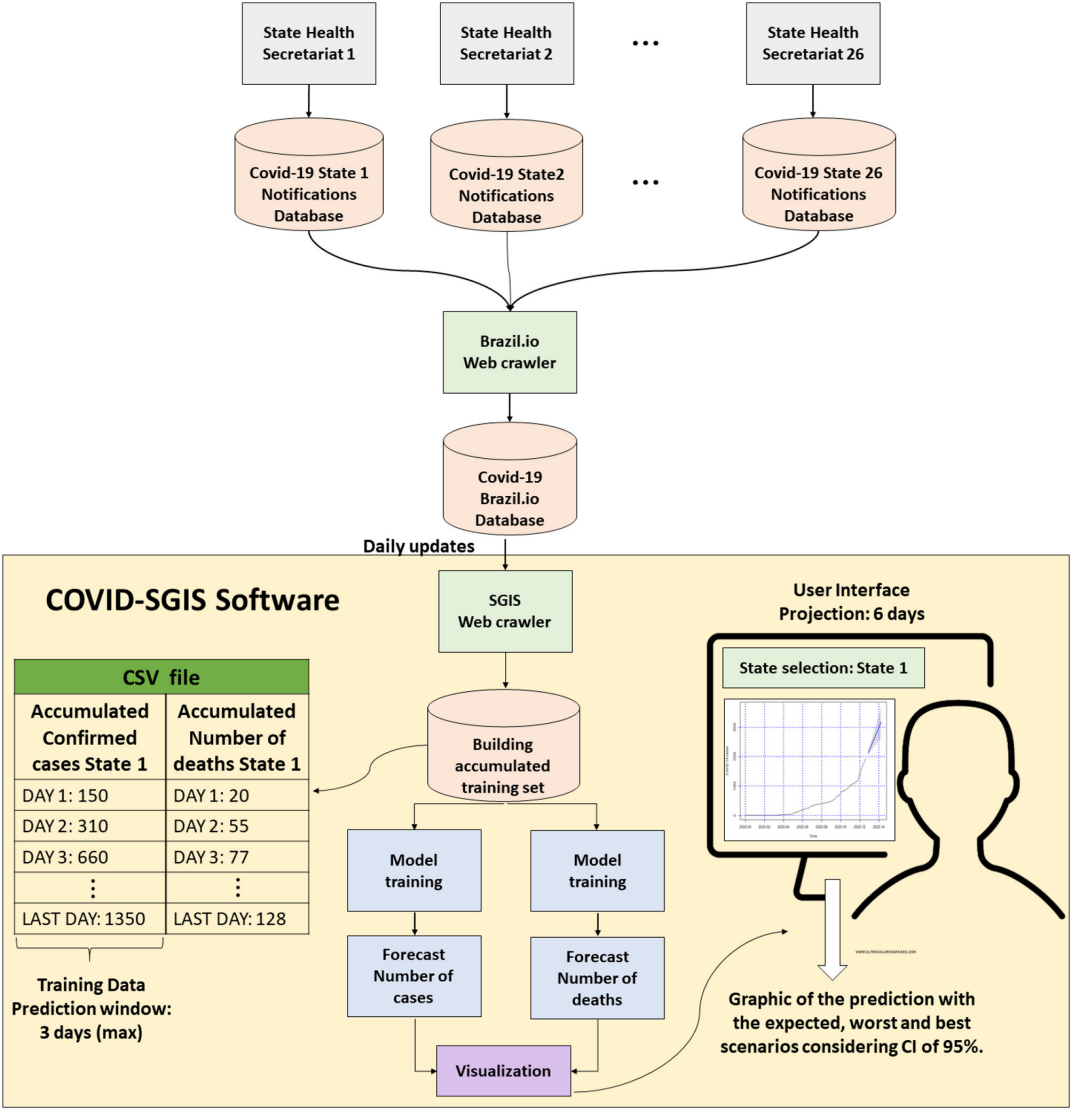
Proposed method: Each of the Health Secretariat of the 26 Brazilian states plus the Distrito Federal (the autonomous district in which is inserted the national capital) is responsible for feeding a notification base. All of this information is available on Brazil.io. Our Covid-SGIS software is updated daily with data from Brazil.io. A file in CSV format is organized with the accumulated data. From them, training sets of the model can be formed. After the ARIMA model training, the user can view the forecast of the number of cases and deaths for each of the states, with a 6-days projection.

In this work, we proposed a system for real-time forecast of the cumulative cases of Covid-19 in Brazil, and for each of its 27 federative units (or state). The system operates as follows: Each Brazilian state feeds a database of Covid-19 notifications. All of this information is gathered in a general database, the Brazil.io. Then, our developed software, the COVID-SGIS, captures Covid-19 data which is made readily available by the Brasil.io portal (https://brasil.io/home/) on a daily basis through the SGIS web crawler. This accumulated data are collated into a comma separated value (.csv) file. We can in turn, derive training datasets from this data for which models are created for the accumulated number of confirmed and reported deaths of Covid-19. With the generated models, 6-days forecasts are reported with their corresponding 95% CI. The forecasted graphs of the accumulated confirmed cases and deaths caused by Covid-19 are available for Brazil and for each of its federative units, separately. In addition to the forecasts, the worst and best scenarios are presented for the number of confirmed cases as well as the number of deaths by Covid-19. Figure 2 shows the general schematic of this solution.

### 3.2. Confirmed Cases Database

We used the data referring to confirmed cases available in the Brasil.io portal^1^ in our temporal forecast approach. Brasil.io portal provides the records of confirmed cases and deaths obtained through the bulletins of the State Health Secretariats. In this work, we selected data related to confirmed cases of Covid-19 for all Brazilian federative units. For each federative unit (out of 27), the records is from the first date of confirmation of the disease until the last update on May 5, 2020.

### 3.3. ARIMA Model

ARIMA models are classic models widely used to forecast future behaviors of stationary time series. ARIMA's acronym indicates the combination of autoregressive (AR) and moving average (MA) models. The “I” which stands for “integrated,” this indicates the model's original undifferenced series, which was differenced *d* times until it became a stationary series before fitting the ARMA(p,q) process. In the ARIMA(p, d, q) model, *p* and *q* correspond to the orders of the AR and MA models, respectively, while the *d* corresponds to the level of differencing. Equation (17) represents the mathematical expression of the model, where *y*_*t*_ is the differenced series, *c* and ϕ are the parameters of the model and ε is the random error in time *t* (27, 28).

(17) $$\begin{array}{l} y_{t}^{\prime }=c+\phi_{1}y_{t-1}^{\prime }+...+\phi_{p}y_{t-p}^{\prime }+\theta_{1}\varepsilon_{t-1}+...+\theta_{q}\varepsilon_{t-q}+\varepsilon_{t} \end{array}$$

The construction of the ARIMA model (p, d, q) occurs according to the following steps (27, 28):

1. Evaluate the stationarity of the series (if it is not stationary, the differencing is applied until the stationarity is achieved). The results of the Dickey-Fuller test are shown in Table 1.
2. Estimate the *p* and *q* parameters based on the autocorrelation function (ACF) and the partial autocorrelation function (PACF) plots.
3. Evaluate the best fitted forecasting model using Akaike Information Criterion (AIC) and the Bayesian Information Criterion (BIC).

**Table 1:** Results of the Dickey-Fuller tests of the historical series of the accumulated number of cases of covid-19 in Brazil and in its 27 federative units.

**State**	**Differencing order**	***t*-statistics**	**τ**
Acre	*d* = 0	4.4121	−1.95
	*d* = 1	1.874	−1.95
	*d* = 2	−3.983	−1.95
Alagoas	*d* = 0	1.6772	−1.95
	*d* = 1	−0.7374	−1.95
	*d* = 2	−7.8526	−1.95
Amazonas	*d* = 0	5.8866	−1.95
	*d* = 1	1.6766	−1.95
	*d* = 2	−5.5631	−1.95
Amapá	*d* = 0	4.674	−1.95
	*d* = 1	−0.2412	−1.95
	*d* = 2	−6.2019	−1.95
Bahia	*d* = 0	7.9516	−1.95
	*d* = 1	1.4916	−1.95
	*d* = 2	−8.651	−1.95
Ceará	*d* = 0	6.1006	−1.95
	*d* = 1	0.7138	−1.95
	*d* = 2	−2.7642	−1.95
Distrito Federal	*d* = 0	5.481	−1.95
	*d* = 1	−1.0392	−1.95
	*d* = 2	−8.9064	−1.95
Espírito Santo	*d* = 0	4.7973	−1.95
	*d* = 1	−1.1292	−1.95
	*d* = 2	−9.1055	−1.95
Goiás	*d* = 0	5.0383	−1.95
	*d* = 1	−0.9949	−1.95
	*d* = 2	−7.6139	−1.95
Maranhão	*d* = 0	4.1517	−1.95
	*d* = 1	0.674	−1.95
	*d* = 2	−5.2222	−1.95
Minas Gerais	*d* = 0	6.9037	−1.95
	*d* = 1	0.62	−1.95
	*d* = 2	−9.5418	−1.95
Mato Grosso do Sul	*d* = 0	3.988	−1.95
	*d* = 1	−1.9052	−1.95
	*d* = 2	−7.5919	−1.95
Mato Grosso	*d* = 0	4.9929	−1.95
	*d* = 1	−1.1216	−1.95
	*d* = 2	−6.0585	−1.95
Pará	*d* = 0	5.8845	−1.95
	*d* = 1	0.5428	−1.95
	*d* = 2	−5.5288	−1.95
Paraíba	*d* = 0	7.6861	−1.95
	*d* = 1	2.0185	−1.95
	*d* = 2	−7.0357	−1.95
Pernambuco	*d* = 0	3.5281	−1.95
	*d* = 1	−0.2507	−1.95
	*d* = 2	−8.4942	−1.95
Piauí	*d* = 0	5.693	−1.95
	*d* = 1	1.7041	−1.95
	*d* = 2	−5.3715	−1.95
Paraná	*d* = 0	2.5805	−1.95
	*d* = 1	−1.4351	−1.95
	*d* = 2	−7.2115	−1.95
Rio de Janeiro	*d* = 0	8.1792	−1.95
	*d* = 1	0.3303	−1.95
	*d* = 2	−9.0729	−1.95
Rio Grande do Norte	*d* = 0	4.6418	−1.95
	*d* = 1	−1.7051	−1.95
	*d* = 2	−6.9444	−1.95
Rondônia	*d* = 0	4.8637	−1.95
	*d* = 1	0.3534	−1.95
	*d* = 2	−5.8114	−1.95
Roraima	*d* = 0	3.0461	−1.95
	*d* = 1	0.1819	−1.95
	*d* = 2	−2.3616	−1.95
Rio Grande do Sul	*d* = 0	4.1479	−1.95
	*d* = 1	0.3525	−1.95
	*d* = 2	−3.7048	−1.95
Santa Catarina	*d* = 0	3.7958	−1.95
	*d* = 1	−1.6657	−1.95
	*d* = 2	−8.1044	−1.95
Sergipe	*d* = 0	8.3063	−1.95
	*d* = 1	−0.2861	−1.95
	*d* = 2	−12.0538	−1.95
São Paulo	*d* = 0	4.3266	−1.95
	*d* = 1	−1.4522	−1.95
	*d* = 2	−7.4511	−1.95
Tocantis	*d* = 0	5.9875	−1.95
	*d* = 1	1.1544	−1.95
	*d* = 2	−7.4744	−1.95
Brazil	*d* = 0	5.1648	−1.95
	*d* = 1	1.4947	−1.95
	*d* = 2	−5.7441	−1.95

*When t-statistic is greater than τ, it indicates that the series has a single root (it is non-stationary). When t-statistic is lower than τ, the series is stationary*.

The function *auto.arima()* in the forecast package in R, automatically calculates the *p*, *d* and *q* parameters and returns a fitted model. The *forecast()* function of this same package can be used to forecast based on the model adjusted by the *auto.arima()* function (21). In this context, from the data collected regarding the accumulated number of confirmed cases by state, we created a database corresponding to the historical series of accumulated confirmed cases of Covid-19 for Brazil and each of its federative units. We considered the period from the first notification date of the disease until May 5, 2020. From this historical series of accumulated cases of Covid-19. To this end, we built three types of ARIMA models: (1) one which was stratified by each state; (2) another stratified by Distrito Federal; and lastly, (3) one for the overall Brazil. To reiterate, these models were generated using the function *auto.arima()* from forecast package in R (version 3.6.3)^2^. Each model built was used to carry out the projection of the accumulated cases of the disease for 6 days, with a confidence interval of 95%. In order to evaluate the performance of the forecasts, we used accumulated data from May 6 to 11, which were updated on May 13.

### 3.4. Metrics

We selected two metrics to evaluate the models: the correlation coefficient and the Relative Quadratic Error (RMSE percentage). The correlation coefficient is a statistical measure between expected and forecasted values. This value varies from −1 to 1. When it approaches 1, it indicates a strong positive correlation. Conversely, when the correlation coefficient is close to −1, it indicates that the variables have a strong negative correlation. Of course, when the correlation coefficient is close to zero, it indicates that there is no correlation between the variables (29). The value of the correlation coefficient serves as the global evaluator for the model—thus, it is possible to obtain a high correlation coefficient as well as at the same time obtain high values for local errors. For this reason, it cannot be the only metric for assessing model performance. In order to avoid a superficial evaluation of the regressors, we therefore chose the RMSE (%) as an evaluation metric. Equation (18) shows the expression of the calculation of the relative quadratic error, where *p* is the forecasted value and *a* is the actual value.

(18) $$\begin{array}{l} \text{RMSE}\left(\%\right)=\sqrt{\frac{{\left(p_{1}-a_{1}\right)}^{2}+...+{\left(p_{n}-a_{n}\right)}^{2}}{{\left(a_{1}-a_{m}\right)}^{2}+...+{\left(a_{n}-a_{m}\right)}^{2}}}\times 100\% \end{array}$$

In addition to the RMSE(%), we also calculated the Root Mean Square Error (RMSE), the Mean Absolute Error (MAE), the Mean Absolute Percentage Error (MAPE), and the Mean Percentage Error (MPE) (Equations 19–22):

(19) $$\begin{array}{l} \text{RMSE}=\sqrt{\frac{1}{n}\overset{n}{\underset{t=1}{\sum }}{e_{t}}^{2}}, \end{array}$$

(20) $$\begin{array}{l} \text{MAE}=\frac{1}{n}\overset{n}{\underset{t=1}{\sum }}|e_{t}|, \end{array}$$

(21) $$\begin{array}{l} \text{MAPE}=\frac{100\%}{n}\overset{n}{\underset{i=1}{\sum }}\left|\frac{e_{t}}{a_{t}}\right|, \end{array}$$

(22) $$\begin{array}{l} \text{MPE}=\frac{100\%}{n}\overset{n}{\underset{i=1}{\sum }}\frac{f-a}{f}, \end{array}$$

where, *f* is the forecasted value, *a* is the actual value and *e* is the difference between the actual value and the forecasted value.

## 4. Results

**Figure 3:**
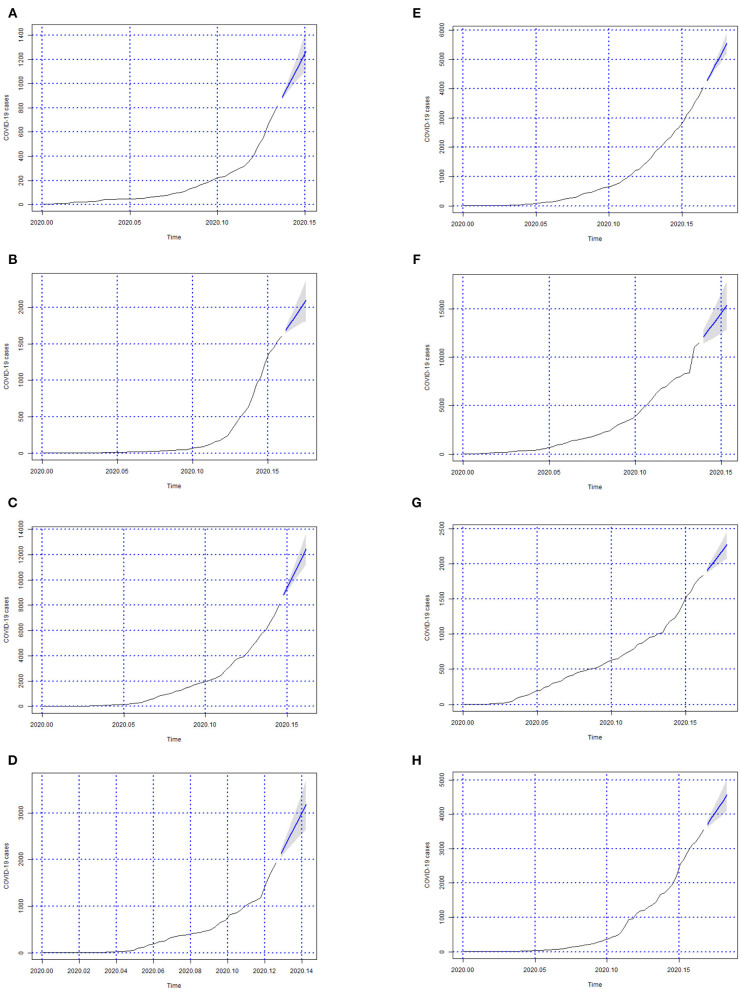
Forecasts of the number of Covid-19 cases from 06-05-2020 to 11-05-2020 for states **(A)** Acre, **(B)** Alagoas, **(C)** Amazonas, **(D)** Amapá, **(E)** Bahia, **(F)** Ceará, **(G)** Distrito Federal, and **(H)** Espírito Santo.

**Figure 4:**
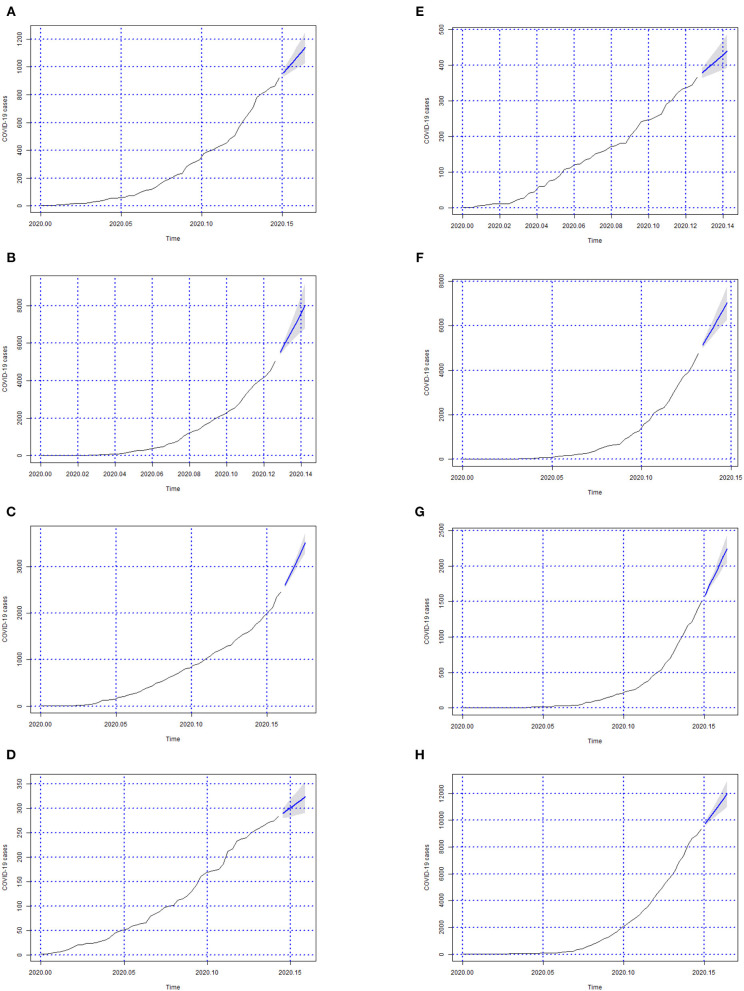
Forecasts of the number of Covid-19 cases from 06-05-2020 to 11-05-2020 for states **(A)** Goiás, **(B)** Maranhão, **(C)** Minas Gerais, **(D)** Mato Grosso do Sul, **(E)** Mato Grosso, **(F)** Pará, **(G)** Paraíba, and **(H)** Pernambuco.

**Figure 5:**
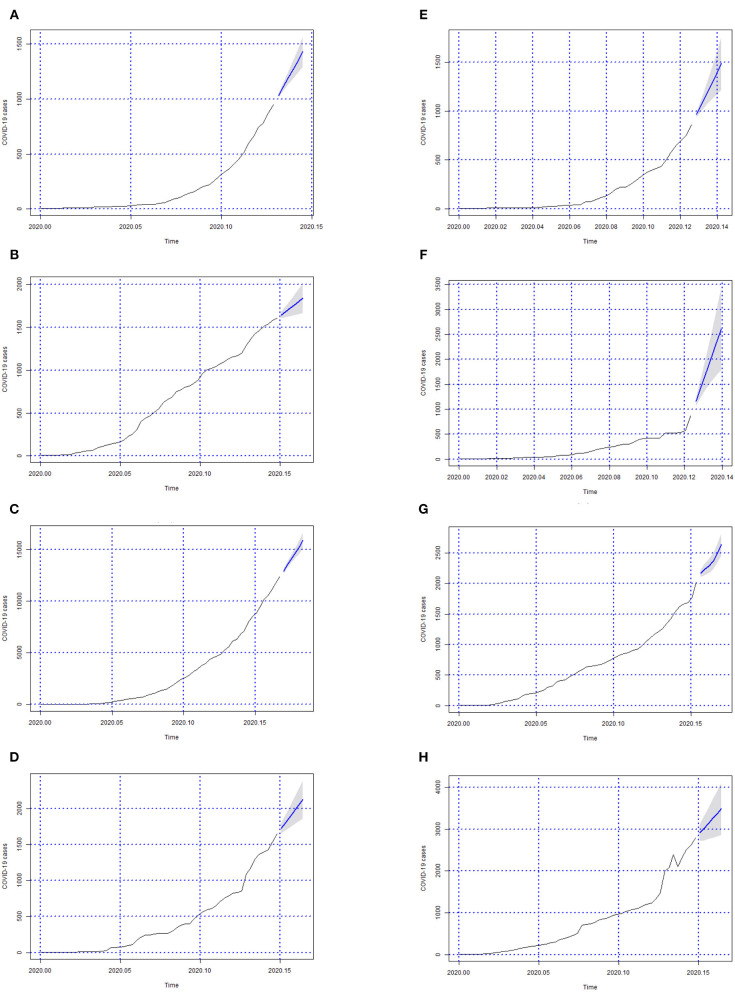
Forecasts of the number of Covid-19 cases from 06-05-2020 to 11-05-2020 for states **(A)** Piauí, **(B)** Paraná, **(C)** Rio de Janeiro, **(D)** Rio Grande do Norte, **(E)** Rondônia, **(F)** Roraima, **(G)** Rio Grande do Sul, and **(H)** Santa Catarina.

**Figure 6:**
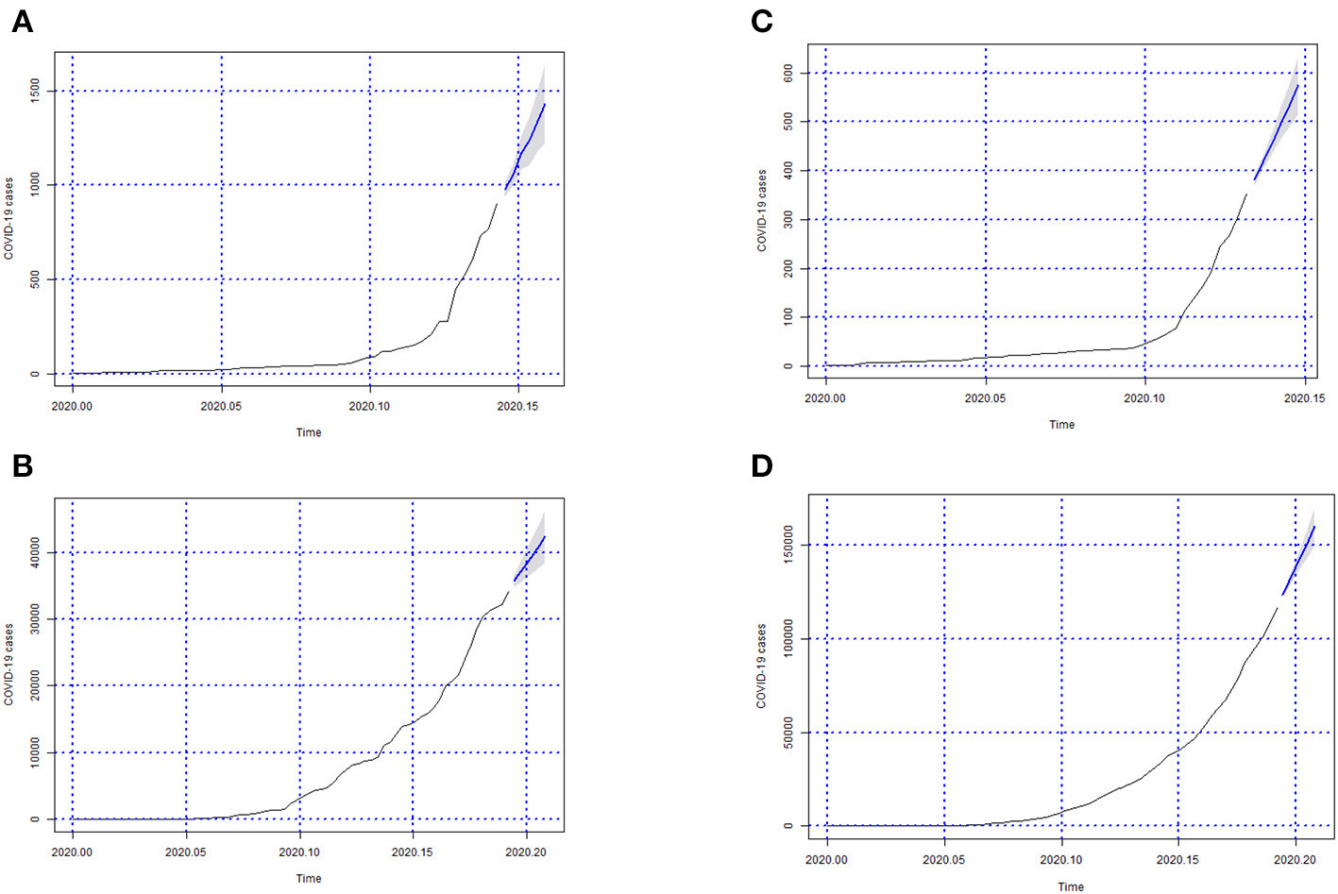
Forecasts of the number of Covid-19 cases from 06-05-2020 to 11-05-2020 for states **(A)** Sergipe, **(B)** São Paulo, **(C)** Tocantins, and **(D)** the whole country.

In Figures 3–6 we can see the forecasts (6 days) of the number of Covid-19 cases for all Brazilian States and the whole country (from 06-05-2020 to 11-05-2020).

### 4.1. ARIMA Forecasting

The models built were evaluated by taking into account, as global quality, the correlation coefficients of Pearson, Spearman, and Kendall. The RMSE (%) was used with a local quality metric. In this work, a high correlation coefficient is considered to be above 0.9 and a low RMSE below 5%. Table 2 shows the evaluation metrics of the results for the models using ARIMA.

**Table 2:** Results of the correlation coefficients of Pearson, Spearman, and Kendall, and of the RMSE% for the ARIMA models built for Brazil and its 27 federative units.

**State**	**Model**	**Pearson**	**Spearman**	**Kendall**	**RMSE**	**RMSE (%)**	**MAPE**	**MPE**	**MAE**	**ME**	**Number of observations**
Acre	ARIMA(2,2,0)	0.99890	0.99515	0.96645	9.30	4.78	7.50	1.48	6.34	1.56	50
Alagoas	ARIMA(0,2,1)	0.99873	0.99371	0.96337	22.21	5.13	6.93	2.36	10.83	2.50	59
Amazonas	ARIMA(0,2,1)	0.99928	0.99899	0.99194	87.65	4.10	6.56	3.77	54.04	20.92	54
Amapá	ARIMA(0,2,1)	0.99647	0.99826	0.98327	43.04	8.78	10.59	3.77	21.64	8.31	47
Bahia	ARIMA(2,2,1)	0.99971	0.99892	0.98795	28.59	2.56	7.78	1.30	18.40	7.36	61
Ceará	ARIMA(0,2,1)	0.99408	1.00000	1.00000	351.5	11.31	5.69	4.65	122.61	75.44	51
Distrito Federal	ARIMA(0,2,1)	0.99922	0.99937	0.99151	20.70	4.02	7.00	3.56	14.29	3.87	60
Espírito Santo	ARIMA(0,2,1)	0.99874	0.99870	0.98565	51.50	5.09	7.82	3.70	26.9	8.60	62
Goiás	ARIMA(0,2,1)	0.99884	0.99971	0.99596	13.55	4.91	5.50	2.62	8.54	2.67	55
Maranhão	ARIMA(0,2,0)	0.99908	0.99867	0.98841	63.78	4.42	10.00	1.20	41.77	10.57	47
Minas Gerais	ARIMA(1,2,2)	0.99945	0.99982	0.99766	24.16	3.46	6.88	3.40	15.64	4.57	59
Mato Grosso do Sul	ARIMA(0,2,1)	0.99860	0.99966	0.99600	4.88	5.40	5.06	2.37	3.36	0.78	53
Mato Grosso	ARIMA(0,2,1)	0.99828	0.99841	0.98654	6.76	6.03	7.75	3.54	4.96	1.58	47
Pará	ARIMA(0,2,1)	0.99868	0.99941	0.99191	71.56	5.52	9.5	4.44	41.76	19.46	49
Paraíba	ARIMA(3,2,0)	0.99931	0.99586	0.97577	15.11	3.91	13.92	3.37	8.52	3.42	55
Pernambuco	ARIMA(0,2,1)	0.99949	0.99955	0.99360	89.79	3.28	7.58	3.08	48.25	19.18	55
Piauí	ARIMA(1,2,0)	0.99928	0.99813	0.98266	10.28	4.00	7.2	1.12	6.41	2.55	48
Paraná	ARIMA(0,2,2)	0.99942	0.99949	0.99392	18.18	3.47	4.96	2.90	12.96	2.67	55
Rio de Janeiro	ARIMA(0,2,2)	0.99960	0.99918	0.99258	105.29	2.96	8.78	2.44	67.51	27.06	62
Rio Grande do Norte	ARIMA(0,2,1)	0.99741	0.99727	0.97657	34.52	7.37	8.24	4.36	18.31	6.99	55
Rondônia	ARIMA(0,2,0)	0.99819	0.99485	0.96633	14.40	6.20	10.61	−0.09	7.98	2.19	47
Roraima	ARIMA(0,2,0)	0.97919	0.99321	0.94769	43.73	21.08	12.08	1.56	19.02	6.33	46
Rio Grande do Sul	ARIMA(2,2,2)	0.99882	0.99940	0.99278	27.95	5.10	7.56	2.71	17.73	6.35	57
Santa Catarina	ARIMA(0,2,1)	0.99254	0.99955	0.99562	96.88	12.41	6.72	4.25	38.95	17.18	55
Sergipe	ARIMA(3,2,0)	0.99598	0.99631	0.97041	18.97	9.11	9.84	1.39	7.93	3.22	53
São Paulo	ARIMA(0,2,2)	0.99917	0.99914	0.98688	411.73	4.14	9.96	4.26	243.34	74.83	71
Tocantins	ARIMA(0,2,2)	0.99785	0.99308	0.95766	5.52	6.76	10.02	2.40	3.07	1.20	49
Brazil	ARIMA(0,2,1)	0.99978	0.99718	0.97963	688.24	2.20	15.65	−0.06	401.29	142.02	71

Tables 3–**7**, present the results for the forecast using the ARIMA models for Brazil and each of its 27 federative units. The performance of the models was evaluated by taking into account erro quadrático médio de between the forecasted values and the actual cases of the accumulated cases of Covid-19, as well as the absolute error between the actual cases and the lower or limit of the forecast. The values highlighted in red represent the situations in which the value of the actual cases are outside of the forecasted interval and the absolute error in between the lower or upper limit is <5%.

**Table 3:** Results of projections of confirmed cases of Covid-19 between May 6 and 11, 2020 for the states of the Northeast Region.

**State**	**Date**	**Forecasted value**	**Lower limit**	**Upper limit**	**Actual cases**	Absolute percentage error*	Absolute percentage error**	**MAPE**
Alagoas	2020-05-06	1,687	1,642	1,732	1,703	0.95	–	7.91
	2020-05-07	1,769	1,686	1,851	1,867	5.27	0.84	
	2020-05-08	1,851	1,725	1,976	2,033	8.97	2.81	
	2020-05-09	1,932	1,759	2,105	2,170	10.95	2.98	
	2020-05-10	2,014	1,789	2,239	2,258	10.80	0.83	
	2020-05-11	2,096	1,815	2,377	2,343	10.54	–	
Bahia	2020-05-06	4,288	4,230	4,347	4,301	0.29	–	2.24
	2020-05-07	4,515	4,416	4,614	4,528	0.29	–	
	2020-05-08	4,821	4,672	4,969	4,818	0.05	–	
	2020-05-09	5,023	4,807	5,239	5,174	2.91	–	
	2020-05-10	5,320	5,044	5,596	5,558	4.28	–	
	2020-05-11	5,555	5,200	5,910	5,808	4.35	–	
Ceará	2020-05-06	12,119	11,409	12,829	12,310	1.55	–	9.66
	2020-05-07	12,768	11,677	13,858	13,888	8.07	0.21	
	2020-05-08	13,416	11,973	14,860	15,134	11.35	1.81	
	2020-05-09	14,065	12,272	15,858	15,879	11.42	0.13	
	2020-05-10	14,714	12,567	16,861	16,692	11.85	–	
	2020-05-11	15,363	12,854	17,872	17,599	12.71	–	
Maranhão	2020-05-06	5,526	5,398	5,654	5,389	2.54	–	5.18
	2020-05-07	6,024	5,738	6,310	5,909	1.95	–	
	2020-05-08	6,522	6,044	7,000	6,765	3.59	–	
	2020-05-09	7,020	6,320	7,720	7,599	7.62	–	
	2020-05-10	7,518	6,571	8,465	8,144	7.69	–	
	2020-05-11	8,016	6,797	9,235	8,526	5.98	–	
Paraíba	2020-05-06	1,586	1,555	1,617	1,657	4.26	2.39	7.11
	2020-05-07	1,750	1,701	1,799	1,849	5.35	2.68	
	2020-05-08	1,860	1,782	1,938	2,030	8.39	4.55	
	2020-05-09	1,987	1,870	2,104	2,156	7.85	2.43	
	2020-05-10	2,136	1,983	2,289	2,341	8.75	2.21	
	2020-05-11	2,243	2,044	2,443	2,525	11.15	3.26	
Pernambuco	2020-05-06	9,767	9,586	9,948	9,881	1.16	–	8.69
	2020-05-07	10,208	9,894	10,522	10,824	5.69	2.79	
	2020-05-08	10,650	10,193	11,107	11,587	8.09	4.14	
	2020-05-09	11,092	10,479	11,705	12,470	11.05	6.14	
	2020-05-10	11,534	10,753	12,314	13,275	13.12	7.24	
	2020-05-11	11,975	11,015	12,935	13,768	13.02	6.05	
Piauí	2020-05-06	1,032	1,012	1,053	1,051	1.78	–	1.47
	2020-05-07	1,111	1,074	1,147	1,131	1.81	–	
	2020-05-08	1,192	1,133	1,250	1,233	3.36	–	
	2020-05-09	1,271	1,188	1,354	1,278	0.54	–	
	2020-05-10	1,351	1,242	1,461	1,332	1.45	–	
	2020-05-11	1,431	1,292	1,570	1,443	0.82	–	
Rio Grande do Norte	2020-05-06	1,724	1,654	1,794	1,739	0.86	–	3.42
	2020-05-07	1,804	1,695	1,913	1,821	0.93	–	
	2020-05-08	1,884	1,737	2,031	1,919	1.82	–	
	2020-05-09	1,964	1,779	2,149	1,919	2.35	–	
	2020-05-10	2,044	1,819	2,269	1,919	6.52	–	
	2020-05-11	2,124	1,858	2,390	1,989	6.79	–	
Sergipe	2020-05-06	977	938	1,016	998	2.08	–	17.64
	2020-05-07	1,055	999	1,111	1,214	13.12	8.52	
	2020-05-08	1,171	1,083	1,260	1,438	18.55	12.39	
	2020-05-09	1,236	1,108	1,363	1,588	22.18	14.16	
	2020-05-10	1,341	1,178	1,504	1,771	24.29	15.09	
	2020-05-11	1,431	1,220	1,642	1,800	20.49	8.77	

The * symbol is the error between the forecasted values and the current cases, while ** indicates the percentage error between the current value and one of the limits (lower or higher).

The findings in Table 3 show the resulting outputs from the ARIMA models for the Northeast Region of Brazil. For the states of Bahia, Maranhão, Piauí, and Rio Grande do Norte, the actual cases were within the range of the forecast limits from 06 to 11 May. The ARIMA model for the State of Piauí showed the best performance. The errors obtained for actual and estimated cases substantially varied from 0.54 to 3.36%. On the other hand, the model that had the worst performance for this region was the State of Sergipe. For that state in particular and from its 6 days of forecast, we observed that the cases estimated for May 6 were within the forecast interval. On the remaining days, we found that the actual cases accumulated for Covid-19 exceeded the maximum limits of the projections, with errors of 8.52, 12.39, 14.16, 15.09, and 8.77% (see absolute percentage error of Sergipe in Table 2).

Table 4 show the forecasted findings for all states in the Brazilian Northern Region. The models that presented the best performance were the ones for the states of Amapá and Rondônia. For the 6 days of forecast, the actual cases were within the minimum and maximum limits. The worst performances were for the states of Roraima and Tocantins. For those states, the errors between the actual cases and the minimum and maximum limits, respectively, reached more than 55 and 25%.

**Table 4:** Results of projections of confirmed cases of covid-19 between May 6 and 11, 2020 for the states of the Northern Region.

**State**	**Date**	**Forecasted value**	**Lower limit**	**Upper limit**	**Actual cases**	Absolute percentage error*	Absolute percentage error**	**MAPE**
Acre	2020-05-06	889	870	908	943	5.73	3.69	11.48
	2020-05-07	968	931	1,005	1,014	4.54	0.90	
	2020-05-08	1,041	977	1,105	1,177	11.56	6.13	
	2020-05-09	1,118	1,025	1,211	1,335	16.26	9.27	
	2020-05-10	1,192	1,064	1,320	1,447	17.63	8.77	
	2020-05-11	1,268	1,102	1,434	1,460	13.16	1.81	
Amazonas	2020-05-06	8,833	8,656	9,010	9,243	4.43	–	5.38
	2020-05-07	9,557	9,218	9,897	10,099	5.36	2.00	
	2020-05-08	10,282	9,754	10,809	10,727	4.15	–	
	2020-05-09	11,006	10,267	11,744	11,925	7.71	1.52	
	2020-05-10	11,730	10,759	12,701	12,599	6.90	–	
	2020-05-11	12,454	11,231	13,677	12,919	3.60	–	
Amapá	2020-05-06	2,140	2,052	2,227	2,046	4.58	–	11.31
	2020-05-07	2,348	2,189	2,508	2,199	6.79	–	
	2020-05-08	2,557	2,316	2,798	2,322	10.11	–	
	2020-05-09	2,765	2,435	3,096	2,493	10.93	–	
	2020-05-10	2,974	2,545	3,403	2,613	13.82	–	
	2020-05-11	3,183	2,647	3,718	2,671	19.16	–	
Pará	2020-05-06	5,136	4,991	5,281	5,524	7.03	4.40	8.65
	2020-05-07	5,516	5,267	5,765	5,935	7.06	2.87	
	2020-05-08	5,896	5,536	6,256	6,519	9.56	4.04	
	2020-05-09	6,276	5,796	6,756	7,018	10.58	3.74	
	2020-05-10	6,656	6,046	7,265	7,348	9.42	1.13	
	2020-05-11	7,035	6,288	7,783	8,069	12.81	3.54	
Rondônia	2020-05-06	966	937	995	943	2.44	–	7.54
	2020-05-07	1,071	1,007	1,135	1,098	2.46	–	
	2020-05-08	1,176	1,068	1,284	1,222	3.76	–	
	2020-05-09	1,281	1,123	1,439	1,263	1.43	–	
	2020-05-10	1,386	1,172	1,600	1,302	6.45	–	
	2020-05-11	1,491	1,216	1,766	1,398	6.65	–	
Roraima	2020-05-06	1,160	1,072	1,248	932	24.46	15.06	62.15
	2020-05-07	1,451	1,255	1,647	1,020	42.25	23.04	
	2020-05-08	1,742	1,414	2,070	1,124	54.98	25.81	
	2020-05-09	2,033	1,553	2,513	1,202	69.13	29.20	
	2020-05-10	2,324	1,674	2,974	1,290	80.16	29.77	
	2020-05-11	2,615	1,779	3,451	1,295	101.93	37.37	
Tocantins	2020-05-06	381	370	392	423	9.90	7.24	23.19
	2020-05-07	420	405	434	494	15.00	12.07	
	2020-05-08	459	437	481	572	19.81	15.97	
	2020-05-09	497	465	530	688	27.70	22.97	
	2020-05-10	536	491	581	747	28.21	22.17	
	2020-05-11	575	516	634	828	30.55	23.37	

The * symbol is the error between the forecasted values and the current cases, while ** indicates the percentage error between the current value and one of the limits (lower or higher).

The forecasted findings for the states of the Midwest Region and the Distrito Federal are presented in Table 5. The models referring to the states of Mato Grosso do Sul and Goiás presented the best performances for the region. In the case of the State of Goiás, only the forecast for May 6 was not within the forecast's interval. However, the absolute percentage error between the actual cases and the upper limit did not exceed the 5% mark. In contrast, for the State of Mato Grosso do Sul, the cumulative sum of the actual cases of Covid-19 was not within the forecast's interval. However, the errors considering the actual cases and the upper limit were not <5%. Meanwhile, both models for the state of Mato Grosso and the Distrito Federal presented a low performance, with the latter showing to be the worst for the region. It should be noted that for the 6 days of forecast, all of them were outside the interval between the lower limit and the upper limit. Additionally, only the cumulative sum of the actual cases for May 6 obtained an error below 5% in relation to the upper limit.

**Table 5:** Results of projections of confirmed cases of Covid-19 between May 6 and 11, 2020 for the states of the Midwest Region of Brazil and the Distrito Federal.

**State**	**Date**	**Forecasted value**	**Lower limit**	**Upper limit**	**Actual cases**	Absolute percentage error*	Absolute percentage error**	**MAPE**
Distrito Federal	2020-05-06	1,910	1,868	1,951	2,046	6.66	4.63	14.81
	2020-05-07	1,982	1,914	2,051	2,258	12.21	9.17	
	2020-05-08	2,055	1,959	2,151	2,442	15.85	11.90	
	2020-05-09	2,128	2,002	2,253	2,576	17.40	12.52	
	2020-05-10	2,200	2,044	2,357	2,682	17.96	12.11	
	2020-05-11	2,273	2,083	2,463	2,799	18.79	12.01	
Goias	2020-05-06	958	931	986	1,024	6.43	3.76	1.77
	2020-05-07	994	951	1,038	1,027	3.18	–	
	2020-05-08	1,031	971	1,090	1,053	2.13	–	
	2020-05-09	1,067	991	1,143	1,069	0.21	–	
	2020-05-10	1,103	1,010	1,196	1,093	0.91	–	
	2020-05-11	1,139	1,028	1,250	1,100	3.56	–	
Mato Grosso do Sul	2020-05-06	290	280	300	288	0.62	–	8.68
	2020-05-07	297	282	311	311	4.64	–	
	2020-05-08	303	284	323	326	6.95	1.00	
	2020-05-09	310	286	334	346	10.37	3.50	
	2020-05-10	317	289	345	362	12.46	4.69	
	2020-05-11	324	291	356	385	15.93	–	
Mato Grosso	2020-05-06	378	365	392	385	1.74	–	14.84
	2020-05-07	391	370	411	419	6.78	–	
	2020-05-08	403	375	430	464	13.17	7.26	
	2020-05-09	415	381	449	502	17.29	10.55	
	2020-05-10	427	387	468	519	17.63	9.87	
	2020-05-11	440	393	487	545	19.31	10.70	

The * symbol is the error between the forecasted values and the current cases, while ** indicates the percentage error between the current value and one of the limits (lower or higher).

The results of the forecasts for the states of the Southeast Region are presented in Table 6. Among the four models generated, only the model from the State of Rio de Janeiro did not show a good performance. From the 6 days of forecast, only two met the criteria of a good performance. For the states of Espírito Santo and Minas Gerais, the forecasts for the 6 days were within the forecast interval. In these two states, the absolute percentage errors between the actual cases and the forecasted values varied between 0.10 and 5.36%, for the State of Espírito Santo, and between 0.03 and 5.74%, for the State of Minas Gerais. The actual cases for the State of São Paulo, for most of the forecast days, were outside the lower limit and upper limit interval. However, for those days (May 6th–10th), the errors between the actual cases and the upper limits ranged from 2.40 to 3.57%.

**Table 6:** Results of projections of confirmed cases of Covid-19 between May 6 and 11, 2020 for the states of the Southeast Region of Brazil.

**State**	**Date**	**Forecasted value**	**Lower limit**	**Upper limit**	**Actual cases**	Absolute percentage error*	Absolute percentage error**	**MAPE**
Espírito Santo	2020-05-06	3,710	3,607	3,814	3,714	0.10	–	3.59
	2020-05-07	3,880	3,709	4,052	3,988	2.70	–	
	2020-05-08	4,050	3,809	4,291	4,242	4.52	–	
	2020-05-09	4,221	3,906	4,535	4,412	4.34	–	
	2020-05-10	4,391	3,998	4,784	4,599	4.53	–	
	2020-05-11	4,561	4,085	5,037	4,819	5.36	–	
Minas Gerais	2020-05-06	2,596	2,547	2,646	2,605	0.34	–	1.93
	2020-05-07	2,760	2,691	2,830	2,770	0.35	–	
	2020-05-08	2,938	2,845	3,031	2,943	0.17	–	
	2020-05-09	3,124	3,000	3,248	3,123	0.03	–	
	2020-05-10	3,315	3,150	3,481	3,237	2.43	–	
	2020-05-11	3,511	3,295	3,727	3,320	5.74	–	
Rio de Janeiro	2020-05-06	12,911	12,697	13,124	13,295	2.89	–	9.30
	2020-05-07	13,506	13,230	13,782	14,156	4.59	2.64	
	2020-05-08	14,101	13,728	14,474	15,741	10.42	8.05	
	2020-05-09	14,696	14,200	15,191	16,929	13.19	10.26	
	2020-05-10	15,291	14,652	15,929	17,062	10.38	6.64	
	2020-05-11	15,885	15,885	15,088	16,683	17,939	7.00	
São Paulo	2020-05-06	35,672	34,841	36,503	37,853	5.76	3.57	8.83
	2020-05-07	37,010	35,483	38,537	39,928	7.31	3.48	
	2020-05-08	38,348	36,214	40,482	41,830	8.32	3.22	
	2020-05-09	39,686	36,958	42,414	44,411	10.64	4.50	
	2020-05-10	41,024	37,694	44,353	45,444	9.73	2.40	
	2020-05-11	42,362	38,416	4,6308	46,131	8.17	–	

The * symbol is the error between the forecasted values and the current cases, while ** indicates the percentage error between the current value and one of the limits (lower or higher).

The ARIMA models generated for the South Region, as shown in Table 7, obtained good results for the forecasts of the cumulative sum of Covid-19 cases for this region. For both the State of Santa Catarina and the State of Paraná, the actual cases were within the forecast interval and very close to the estimated values. The absolute percentage errors between the real cases of the Covid-19 accumulated cases for the two states varied between 0.26 and 3.21%, and 0.19 and 3.30%, respectively. In the case of the forecasts for Rio Grande do Sul, the actual cases of May 8 and 9 are outside the forecast interval range, however the errors between the actual case number and the upper limit were 3.17 and 1.66%, respectively.

**Table 7:** Results of projections of confirmed cases of Covid-19 between May 6 and 11, 2020 for the states of the South Region of Brazil.

**State**	**Date**	**Forecasted cases**	**Lower limit**	**Upper limit**	**Actual cases**	Absolute percentage error*	Absolute percentage error**	**MAPE**
Paraná	2020-05-06	1,643	1,606	1,680	1,647	0.26	–	1.50
	2020-05-07	1,682	1,616	1,748	1,678	0.23	–	
	2020-05-08	1,721	1,629	1,813	1,734	0.75	–	
	2020-05-09	1,760	1,642	1,879	1,809	2.70	–	
	2020-05-10	1,799	1,654	1,944	1,859	3.21	–	
	2020-05-11	1,838	1,666	2,011	1,873	1.85	–	
Santa Catarina	2020-05-06	2,911	2,716	3,107	2,917	0.19	–	2.10
	2020-05-07	3,028	2,735	3,321	3,082	1.75	–	
	2020-05-08	3,144	2,764	3,524	3,205	1.89	–	
	2020-05-09	3,261	2,797	3,724	3,372	3.30	–	
	2020-05-10	3,377	2,831	3,923	3,429	1.51	–	
	2020-05-11	3,494	2,865	4,123	3,529	1.00	–	
Rio Grande do Sul	2020-05-06	2,167	2,109	2,224	2,100	3.17	–	4.94
	2020-05-07	2,233	2,138	2,328	2,182	2.35	–	
	2020-05-08	2,283	2,166	2,401	2,493	8.41	3.71	
	2020-05-09	2,367	2,233	2,500	2,542	6.90	1.66	
	2020-05-10	2,492	2,340	2,645	2,576	3.25	–	
	2020-05-11	2,640	2,458	2,821	2,808	6.00	–	

The * symbol is the error between the forecasted values and the current cases, while ** indicates the percentage error between the current value and one of the limits (lower or higher).

The results of the forecasts for Brazil are shown in Table 8. From May 6th to 10th, the actual cases were outside the forecast range (they were higher than the upper limit). However, the absolute percentage error between the upper limit and the actual cases did not exceed the 5% limit. On May 11, the value of the actual cases was within the forecast interval, showing an error of 5.63% between the forecasted value and the actual cases.

**Table 8:** Result of the projections of the accumulated confirmed cases of Covid-19 for Brazil between May 6th to 11th, 2020.

**State**	**Date**	**Forecasted value**	**Lower limit**	**Upper limit**	**Actual cases**	Absolute percentage error*	Absolute percentage error**	**MAPE**
Brazil	2020-05-06	123,706	122,328	125,085	126,957	2.56	1.47	5.18
	2020-05-07	131,001	128,154	133,747	136,689	4.16	2.15	
	2020-05-08	138,295	133,946	142,644	147,093	5.98	3.02	
	2020-05-09	145,589	139,424	151,755	155,329	6.27	2.30	
	2020-05-10	152,883	144,709	161,057	163,509	6.50	1.50	
	2020-05-11	160,178	149,819	170,537	169,733	5.63	0.47	

The * symbol is the error between the forecasted values and the current cases, while ** indicates the percentage error between the current value and one of the limits (lower or higher).

### 4.2. Web Application

**Figure 7:**
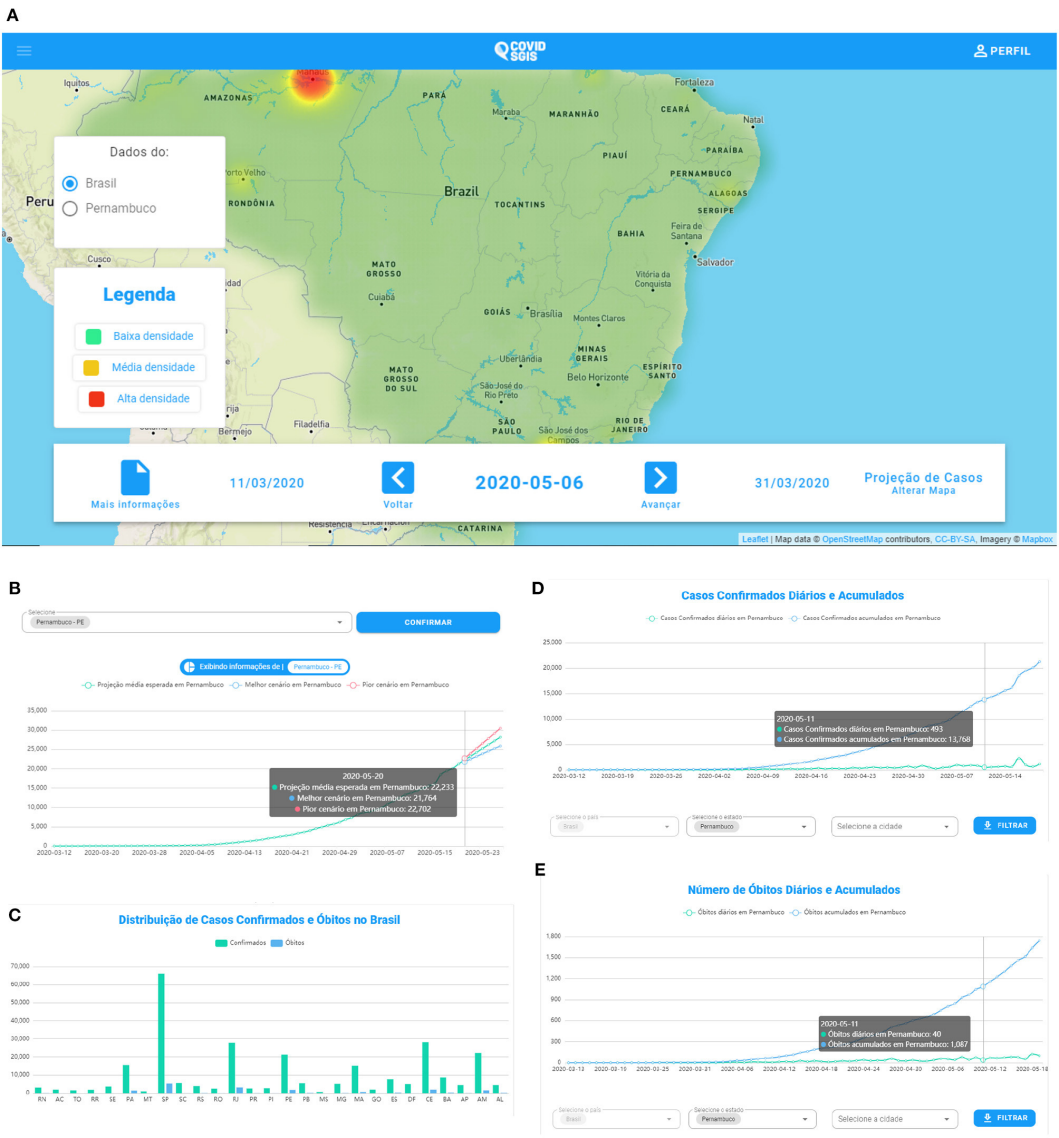
**(A)** COVID SGIS web application home screen. **(B)** Accumulated cases of Covid-19 forecast graph. The forecast with ARIMA is represented by the green line. The worst case scenario (indicated by the upper limit of the forecast) is represented by the line in red. The best scenario (indicated by the lower limit of the forecast) is represented by the blue line. **(C)** Screen of the graph of the distribution of confirmed cases and deaths by Covid-19. In this graph the user can have an overview of the accumulated confirmed cases and death cases in all states of Brazil and the Distrito Federal. In COVID SGIS, the user can follow the daily and accumulated confirmed cases **(D)** and deaths **(E)** of Covid-19 for each Brazilian state and the Distrito Federal, separately.

The prototype of the developed system can be accessed by the link (https://www.cin.ufpe.br/covidsgis). On the home screen, it is possible to visualize information about spatial forecasts (Figure 7A). The projections of the cumulative cases of Covid-19 can be accessed in the “More information” option, in which the user will be directed to the page with the graphics. In this screen, graphs of the temporal forecasts (Figure 7B), as well as graphs referring to the distribution of daily cases and deaths in Brazil (Figure 7C) are also available. In addition, it is available daily and cumulative cases (Figure 7D), as well as daily and cumulative deaths (Figure 7E). For these graphs, the user can select from which state they wish to evaluate these information. The software's backend is freely available for non-commercial purpose on our Github repository: https://github.com/Biomedical-Computing-UFPE/Covid-SGIS.

## 5. Discussion

In this study, we evaluated the forecast of the cumulative cases of Covid-19 for Brazil, and its 27 federative units in Brazil, using ARIMA models in the period between May 6 and 11. To our knowledge, this is the first study to integrate ARIMA models, GIS and data health sciences into a computerized system for the forecast and surveillance of Covid-19 within a Brazilian context. Our findings showed that the overall cases of Covid-19 in Brazil were on the rise during the observed periods, as shown in Figure 6D, whereby such rises were confirmed through the analyses of actual Covid-19 cases for the same time period as shown in Table 8 which shows evidence of the growth in the number of cases. Furthermore, our approach is able to detect the peaks of the disease within this period as shown in Table 8, which was not possible to observe in prior studies.

In Figures 3–6; the images show the forecasts' curves of Covid-19 cases for each of the states in those observed period and how they all tended to increase. This fact was confirmed with the results in the section 4. Both for Brazil and its federative units, the ARIMA(p, d, q) models managed to capture the trend patterns in the curves. This can be useful for health managers and governments in promoting public policies for combating the burden of Covid-19.

One of the limitations of this work is related to the fact that the ARIMA models analyze only univariate time series. Therefore, factors, such as population distribution, the influence of isolation policies and factors linked to population dynamics were not taken into account. Data related to the geographic space and climate of the federative units were also excluded from the model. The authors are aware that this may have contributed to the high errors in the states of Acre, Tocantins and Rondônia.

The low performance of the forecasts for some Brazilian states may be associated with the underreporting of cases. That is, the amount of tests for the diagnosis is not sufficient to meet the populational demand. Thus, people who are asymptomatic or have symptoms of the disease, but have not been tested, are not counted in the reported cases. This problem makes the data substantial underestimated. In addition to this problem linked to underreporting, the authors acknowledge that the forecasting from these models may potentially lack statistical power, a problem which is typically associated with lower sample size which is again due to the undocumented and/or underreported cases in some areas of Brazil.

Another factor that may be associated with higher errors in the forecasts is the time taken to update the databases from the Departments of Health. This directly affects the historical series of the states' accumulated cases, and also the time it takes for the models to process large volumes of data. Indeed, the ARIMA algorithm are quite computationally expensive in terms of time and power needed to crunch large stores of historical data in time series format, and thus such delay in updating the databases are inevitable which, in turn, directly affect the real-time forecasts. In addition, the high forecast errors can also be associated with the historical series itself. As the day of the first notification varies from state-to-state.

Using more features could contribute to improve the forecasts. However, since the main vectors for Covid-19 spreading are the human beings, we believe these new features should emerge from a population model complex enough to include the local population behavior due to economical needs, for instance, since the unemployed population has significantly increased since the beginning of the social distancing measures. Such a population model should be consistent with seasonal variations as well, since these aspects influence economical dynamics in a country as large as Brazil with large environmental diversity. However, since the construction of a sufficient population model to generate additional features for Covid-19 forecasting is out of the scope of this work, we preferred to keep ARIMA due to its computational advantages (essentially, low memory usage and no intensive processing) and relatively good forecasting results in most of cases as discussed above.

Brazil's Covid-19 epidemiological data are very likely to be underestimated. Since 2018, the country has been experiencing a real plague of false news that circulates freely on social networks, especially among people with lower education. This aspect, as well as the action of negationist movements, contributes to disorient the population regarding measures of social distancing and contamination prevention (30). Another important aspect is the high level of social inequality and the low sanitary conditions that affect the lowest income population. According to Prado (30) 13 million Brazilians live in Favelas (i.e., urban settlements which are under-developed and greatest levels of socioeconomic deprivation), where its widespread to see a single room inhabited by more than three people, and where access to clean water and security are precarious. These are factors which we were unable to include in our ARIMA models for Covid-19 due to such paucity of data also due to limitations of modeling with ARIMA. The authors therefore acknowledge the lack of these risk factors and its inclusion to the model may have led to some residual confounding in our analysis.

## 6. Conclusion

Several countries are greatly affected by the increased burden of Covid-19. With a high number of infected people and the public health systems operating at maximum capacity, the situation is becoming increasingly critical. For this reason, it is important to have a tool that performs the forecast of Covid-19 cases so as able support government officials, health managers, and general stakeholders to be informed to execute health policies and targeted interventions. Therefore, it is possible to make short-term decisions, develop public policies, and direct resources to health professionals and hospitals. In addition, the forecasting tool can assist governments in controlling measures of social isolation and lockdown. The evaluation of these forecasts allows to intensify the restrictions of social agglomerations, as well as to evaluate the effect of these measures in the contagion of the population.

With that in mind, our motivation is to provide a robust, flexible, and rapid forecasting method. Thus, we combined the ARIMA model for time series analysis with Artificial Intelligence techniques. ARIMA was an excellent choice for the purpose, being efficient in forecasting Covid-19, which has a rapid proliferation and changes to the daily scenarios. The method is capable of presenting the forecast if the context of the forecast moment remains constant, as well as presenting the best and worst scenarios, with their 95% confidence interval limits.

The developed models achieved good performance when taking into account their percentage errors. Among the 26 Brazilian states analyzed plus Distrito Federal, the majority presented satisfactory results, with low error rates. Some states, such as Roraima, Tocantins, and Distrito Federal showed higher errors. Some hypotheses can be raised for this: many cases of Covid-19 are not reported, as in cases of asymptomatic people or who remained in isolation at home; in addition, database updates can be slow, directly affecting the forecast of the following days. This fact becomes even worse when taking into account the measures of the Brazilian Ministry of Health to limit testing to severe cases attested by clinical diagnosis.

Besides that, some specific issues in Brazil directly affected the course of the pandemic. One fundamental challenge resides on the strong social inequality in the country (31), and the lack of government planning to deal with this reality (32). Many Brazilian citizens do not have a fixed income and a formal job (33). Furthermore, the impact of the pandemic on income-generating activities is most severe for unprotected workers and for the most vulnerable groups in the informal economy (33, 34). Although the Federal Government has been providing monthly aid to the most financially affected families, there are several difficulties in accessing this aid. In addition, in many cases, it is not enough to guarantee basic survival needs. It is also important to highlight the lack of access to hygiene information and basic protection items, such as masks and alcohol, by the less favored population (35). As a consequence, these people need to work and thus break the imposed social isolation policies. In this way, there is a considerable portion of the population circulating on the streets, increasing the population susceptible to contamination by SARS-CoV-2.

Despite this, our method provides a possibility of dynamic forecasting: the proposed model is retrained and adapted to the real scenario in a daily basis. The proposed model is trained everyday with a maximum window of 3 days, achieving low errors in most forecasts, and acceptable errors (inside the confidence interval) in the worst scenarios. This forecasting window is dynamic, since it is chosen by automatic ARIMA models. Another advantage of our proposal is the use of multiple databases. In this way, several countries can benefit from this solution, adapting the model to their databases, incorporating dedicated web crawlers, for instance. Our system can be an important tool to guide the course of this pandemic.

The scope of our proposal comprises the forecast of Covid-19 cases in real time. As a case study, the tool developed, COVID-SGIS, was applied to the forecast of cases in Brazil, in each of the 27 units of the Federation, and in each of the 5,570 municipalities. Brazil is a continental country, with a population of 209.5 million people and involving an area larger than that of Western Europe. Every 24 h, the proposed system collects information from the state health departments for each federative unit and each municipality. And every day the model is retrained with all the data obtained since the official start of the pandemic in the country: March 2020. Thus, a model that could provide good accuracy at a low computational cost of training, with a low demand for memory and processing, is a prerequisite for this problem. Among the low computational cost models investigated, the standard ARIMA has met this prerequisite with an accuracy that can be considered very good or acceptable in most cases.

The situation at Covid-19 is somewhat different from that of other diseases. In arboviruses, for example, vectors and population must be modeled as completely different entities. In addition, the epidemiological dynamics is also influenced by climatic and environmental factors, in addition to socioeconomic factors. In these models, population modeling can help increase accuracy. In the case of Covid-19, we believe that it is quite reasonable to assume that geographic location and the number of cases is the most relevant factors, given that Covid-19 spreads very quickly and that climatic and environmental factors are unrelated to the disease. In Covid-19, the vector and the population coincide, given that the contamination occurs from human to human. The good results obtained by the model we propose show that our hypothesis of focusing on the number of cases, given that the predictors have each municipality and each federative unit as a reference, is correct. Thus, if we wanted to increase the model's accuracy by increasing the number of attributes, these attributes could emerge from a population model. This would be a reasonably complex model, one that could be able to deal with socioeconomic differences that lead to increasing the mobility of low-income populations and to violating social distancing measures. However, there is no guarantee that such a model could contribute to increased accuracy, although this would be an interesting point for future works.

## Data Availability Statement

The datasets referring to confirmed cases of Covid-19 can be found in the Brasil.io website (https://brasil.io/home/). The software's back-end is available on our Github repository: https://github.com/Biomedical-Computing-UFPE/Covid-SGIS.

## Author Contributions

CdL: responsible for the design of forecasting models using machine learning and models' implementation in R code. CdS: responsible for the design of forecasting models using machine learning. AdS, MdS, JG, and VdF: responsible for models' implementation in R code. ES, GM, LdA, LAA, and SSd: front-end and back-end developers. AM, PK, WdS, and AdS: associate researchers and supervisors. All authors contributed to the article and approved the submitted version.

## Conflict of Interest

The authors declare that the research was conducted in the absence of any commercial or financial relationships that could be construed as a potential conflict of interest.

## Footnotes

^1^https://brasil.io/dataset/covid19/caso/

^2^https://www.r-project.org/

## References

[1] Zhang T Wu Q Zhang Z Probable pangolin origin of SARS-CoV-2 associated with the COVID-19 outbreak. Curr Biol. (2020) 30:1346–51.e2. 10.1016/j.cub.2020.03.022 32197085 PMC7156161

[2] WHO Coronavirus Disease (COVID-19) Pandemic. (2020). Available online at: www.who.int/emergencies/diseases/novel-coronavirus-2019 (accessed May 20, 2020).

[3] Guo YR Cao QD Hong ZS Tan YY Chen SD Jin HJ. The origin, transmission and clinical therapies on coronavirus disease 2019 (COVID-19) outbreak-an update on the status. Milit Med Res. (2020) 7:1–10. 10.1186/s40779-020-00240-0 32169119 PMC7068984

[4] Döhla M Boesecke C Schulte B Diegmann C Sib E Richter E Rapid point-of-care testing for SARS-CoV-2 in a community screening setting shows low sensitivity. Public Health. (2020) 182:170–2. 10.1016/j.puhe.2020.04.009 32334183 PMC7165286

[5] Okba NM Muller MA Li W Wang C GeurtsvanKessel CH Corman VM. SARS-CoV-2 specific antibody responses in COVID-19 patients. medRxiv. (2020) 2020. 10.1101/2020.03.18.2003805932267220

[6] Li Z Yi Y Luo X Xiong N Liu Y Li S Development and clinical application of a rapid IgM-IgG combined antibody test for SARS-CoV-2 infection diagnosis. J Med Virol. (2020) 92:1518–24. 10.1002/jmv.25727 32104917 PMC7228300

[7] Liu Y Liu Y Diao B Ren F Wang Y Ding J Diagnostic indexes of a rapid IgG/IgM combined antibody test for SARS-CoV-2. medRxiv. (2020) 2020. 10.1101/2020.03.26.20044883

[8] Chen S Yang J Yang W Wang C Bärnighausen T. COVID-19 control in China during mass population movements at New Year. Lancet. (2020) 395:764–6. 10.1016/S0140-6736(20)30421-9 32105609 PMC7159085

[9] Day M. Covid-19: identifying and isolating asymptomatic people helped eliminate virus in Italian village. BMJ. (2020) 368:m1165. 10.1136/bmj.m1165 32205334

[10] Salathé M Althaus CL Neher R Stringhini S Hodcroft E Fellay J. COVID-19 epidemic in Switzerland: on the importance of testing, contact tracing and isolation. Swiss Med Wkly. (2020) 150:w20225. 10.4414/smw.2020.20225 32191813

[11] Oliveira WKd Duarte E França GVAd Garcia LP. How Brazil can hold back Covid-19. Epidemiol Serv Saúde. (2020) 29:e2020044. 10.5123/S1679-49742020000200023 32348405

[12] Guo P Liu T Zhang Q Wang L Xiao J Zhang Q. Developing a dengue forecast model using machine learning: a case study in China. PLoS Negl Trop Dis. (2017) 11:1–22. 10.1371/journal.pntd.0005973 29036169 PMC5658193

[13] Siriyasatien P Chadsuthi S Jampachaisri K Kesorn K Dengue epidemics prediction: a survey of the state-of-the-art based on data science processes. IEEE Access. (2018) 6:53757–95. 10.1109/ACCESS.2018.2871241

[14] Baquero OS Santana LMR Chiaravalloti-Neto F. Dengue forecasting in São Paulo city with generalized additive models, artificial neural networks and seasonal autoregressive integrated moving average models. PLoS ONE. (2018) 13:e0195065. 10.1371/journal.pone.0195065 29608586 PMC5880372

[15] Wangping J Ke H Yang S Wenzhe C Shengshu W Shanshan Y. Extended SIR prediction of the epidemics trend of COVID-19 in Italy and compared with Hunan, China. Front Med. (2020) 7:169. 10.3389/fmed.2020.00169 32435645 PMC7218168

[16] Bastos S Cajueiro D. Modeling and forecasting the early evolution of the Covid-19 pandemic in Brazil. arXiv. (2020) 200314288. 33173127 10.1038/s41598-020-76257-1 PMC7655855

[17] Liu Z Magal P Seydi O Webb G. Predicting the cumulative number of cases for the COVID-19 epidemic in China from early data. Math Biosci Eng. (2020) 17:3040–51. 10.20944/preprints202002.0365.v1 32987515

[18] Anastassopoulou C Russo L Tsakris A Siettos C. Data-based analysis, modelling and forecasting of the COVID-19 outbreak. PLoS ONE. (2020) 15:e0230405. 10.1371/journal.pone.0230405 32231374 PMC7108749

[19] Yang Z Zeng Z Wang K Wong SS Liang W Zanin M. Modified SEIR and AI prediction of the epidemics trend of COVID-19 in China under public health interventions. J Thorac Dis. (2020) 12:165. 10.21037/jtd.2020.02.64 32274081 PMC7139011

[20] Ardabili SF Mosavi A Ghamisi P Ferdinand F Varkonyi-Koczy AR Reuter U COVID-19 outbreak prediction with machine learning. medRxiv. (2020). 10.31234/osf.io/5dyfc

[21] Hyndman RJ Khandakar Automatic time series forecasting: the forecast package for R. J Stat Softw. (2008) 27:1–22. 10.18637/jss.v027.i03

[22] Kermack W McKendrick A A contribution to the mathematical theory of epidemics. R Soc Lond A Math Phys Sci A. (1927) 115:700–21. 10.1098/rspa.1927.0118

[23] Britton NF. Essencial Mathematical Biology. London: Springer Undergraduate Mathematics Series (2003).

[24] Brauer F Driesseche PWD Wu J Allen LJS Mathematical Epidemiology. Berlin: Springer (1945).

[25] Ma Y Principe JC. A taxonomy for neural memory networks. IEEE Trans Neural Netw Learn Syst. (2020) 31:1780–93. 10.1109/TNNLS.2019.2926466 31443054

[26] Ceylan Z. Estimation of COVID-19 prevalence in Italy, Spain, and France. Sci Total Environ. (2020) 729:138817. 10.1016/j.scitotenv.2020.138817 32360907 PMC7175852

[27] Chakraborty T Gosh I. Real-time forecasts and risk assessment of novel coronavirus (COVID-19) cases: a data-driven analysis. Chaos Solit Fract. (2020) 135:109850. 10.1016/j.chaos.2020.109850 32355424 PMC7190506

[28] Hyndman RJ Athanasopoulos G. Forecasting: Principles and Practice. OTexts (2018).16634120

[29] Witten IH Frank E. Data Mining: Practial Machine Learning Tools and Technique. San Francisco, CA: Morgan Kaufmann Publishers (2005).

[30] Prado B. COVID-19 in Brazil: “so what?”. Lancet. (2020) 395:1461. 10.1016/S0140-6736(20)31095-3 32386576 PMC7251993

[31] Santos JAF COVID-19, fundamental causes, social class and territory. Trabalho Educ Saúde. (2020) 18:1–7. 10.1590/1981-7746-sol00280

[32] Ortega F Orsini M. Governing COVID-19 without government in Brazil: ignorance, neoliberal authoritarianism, and the collapse of public health leadership. Glob Public Health. (2020) 15:1257–7. 10.1080/17441692.2020.1795223 32663117

[33] Costa SdS. Pandemia e desemprego no Brasil. Rev Admin Públ. (2020) 54:969–78. 10.1590/0034-76122020017032785416

[34] Nicola M Alsafi Z Sohrabi C Kerwan A Al-Jabir A Iosifidis C. The socio-economic implications of the coronavirus and COVID-19 pandemic: a review. Int J Surg. (2020) 78:185–93. 10.1016/j.ijsu.2020.04.018 32305533 PMC7162753

[35] Natividade MdS Bernardes K Pereira M Miranda SS Bertoldo J Teixeira MdG. Social distancing and living conditions in the pandemic COVID-19 in Salvador-Bahia, Brazil. Ciênc Saúde Coletiva. (2020) 25:3385–92. 10.1590/1413-81232020259.22142020 32876242

[36] de Lima CL da Silva CC da Silva ACG Silva EL Marques GS de Araujo LJB COVID-SGIS: a smart tool for dynamic monitoring and temporal forecasting of Covid-19. medRxiv. (2020). 10.1101/2020.05.30.20117945 PMC7705350 33282815

